# Expanding the Particle Range for Biocatalytically Active Pickering Emulsions with Silicone Coatings

**DOI:** 10.1002/adma.202508738

**Published:** 2025-07-22

**Authors:** Sara Fatima Bhutta, Christoph Plikat, Marion B. Ansorge‐Schumacher

**Affiliations:** ^1^ Chair of Molecular Biotechnology Dresden University of Technology Zellescher Weg 20b 01217 Dresden Germany

**Keywords:** biocatalyses, microparticles, Pickering emulsions, silicone coatings

## Abstract

Pickering emulsions (PEs) are a particularly promising class of reaction systems for biocatalysis, typically in a water‐in‐oil (w/o) configuration, with a continuous organic phase and a dispersed aqueous phase containing the biocatalyst. However, only a few micro‐ and nanoparticles possess the intrinsic hydrophobicity necessary to stabilize such a w/o PE, which limits the application prospects. Here, silicone coatings are applied to prepare micro particles for w/o PE stabilization for the first time. The study demonstrates compatibility with available inorganic and organic materials, and the effects of silicone coatings with overall hydrophilic, amphiphilic, or hydrophobic properties on the particle size, PE formation, and the stability of the PE. Furthermore, a lipase‐catalyzed transesterification in PE obtained with silicone‐coated particles is performed, and the possibility to place the catalyst not only in the dispersed phase of the PE, but also on the stabilizing particles prior to coating is demonstrated. The results indicate silicone coatings as a straightforward, versatile, and tunable method for the preparation of particles with suitable properties for both the stabilization of BioPE and the immobilization of active biocatalysts.

## Graphical abstract

Pickering emulsions are a promising class of reaction systems for biocatalysis. However, only a few materials can stabilize the required w/o emulsion. In this study, it is demonstrated that coating microparticles with modified silicone is a straightforward, versatile, and tunable method for producing appropriate particles. Consequently, enzyme carriers can be employed as emulsion stabilizers and immobilization materials, thereby intensifying reaction efficiency.

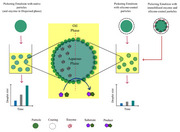

## 1 Introduction

Pickering emulsions (PE) are biphasic emulsion systems stabilized with micro‐ or nano‐sized solid or colloidal particles instead of surfactants.^[^ ^1^, ^2^, ^3^, ^4^, ^5^, ^6^ ^]^ Due to their unparalleled advantages, such as high stability, low toxicity, low cost, environmental friendliness, and flexible, customizable compositions, PEs are important materials for pharmaceuticals, paints and coatings, cosmetics and food industries, medicine, mining, etc.^[^ ^7^, ^8^, ^9^, ^10^, ^11^ ^]^ In recent years, they have also attracted considerable interest as promising reaction systems for biocatalysis and chemocatalysis^[^ ^12^, ^13^, ^14^, ^15^, ^16^, ^17^ ^]^ due to their ability to integrate the advantages of emulsions in combining compounds with different solubility properties with an exceptionally robust stability of the reaction system.^[^ ^18^, ^19^ ^]^ This avoids product contamination and catalyst deactivation^[^ ^20^ ^]^ and benefits catalyst recycling and introduction into multi‐step cascade reactions.^[^ ^12^, ^21^ ^]^

PEs can occur as oil‐in‐water (o/w) or water‐in‐oil (w/o) emulsions, depending on the partial wettability of the stabilizing particles by the water and oil phases, or more simply, the hydrophilicity/hydrophobicity of the particle surface.^[^ ^22^ ^]^ The liquid that predominantly wets the particles forms the continuous phase, and the liquid that wets the particles less forms the dispersed phase. Consequently, particles with a more hydrophobic surface form a w/o PE, whereas particles with a more hydrophilic surface favor the formation of an o/w PE.^[^ ^18^, ^23^ ^]^ The hydrophilicity/hydrophobicity or wettability of the surfaces can be described via the contact angle between the particle surface and water. A contact angle below 90° indicates hydrophilicity and thus o/w PE formation, while a contact angle above 90° demonstrates hydrophobicity and thus a propensity for w/o PE.^[^ ^18^, ^20^ ^]^ Most PE applications currently use the o/w type, but for biocatalytically active PEs (BioPEs) w/o emulsions are typically required, where the biocatalysts are located in the dispersed aqueous phase or on the interface, and the substrates and products are transported over the continuous oil phase.^[^ ^13^, ^15^, ^17^ ^]^

Unfortunately, only a few micro‐ and nanoparticles have demonstrated the intrinsic hydrophobicity to stabilize w/o PE, including carbon‐based materials,^[^ ^3^, ^4^, ^5^, ^22^, ^24^ ^]^ zein (protein) nanoparticles,^[^ ^25^ ^]^ polyphenol crystals,^[^ ^26^ ^]^ and metal–organic‐frameworks (MOFs).^[^ ^27^ ^]^ The majority of other nanoparticles described are initially hydrophilic in nature^[^ ^28^ ^]^ and thus only stabilize o/w type emulsions. To overcome this limitation, a few full particles made from innovative materials were developed, with the focus primarily on soft materials, such as polymer‐based vesicles and colloids^[^ ^25^ ^]^ or modified proteins.^[^ ^29^ ^]^ In most cases, surface modification with small molecules and polymers was used to adapt the wettability of available particles.^[^ ^15^, ^17^, ^28^ ^]^ For example, aluminium oxide particles were enabled to stabilize w/o PE through the adsorption of octyl gallate,^[^ ^30^ ^]^ and magnetic nanoparticles (Fe_3_O_4_) through modification with a carboxylic acid^[^ ^31^ ^]^ or graphene oxide.^[^ ^32^ ^]^ In other studies, calcium‐alginate particles were modified with octyl chains^[^ ^33^ ^]^ or diacetone acrylamide,^[^ ^34^ ^]^ and starch particles with acetic anhydride and phthalic anhydride.^[^ ^35^ ^]^ The vast majority of BioPE currently described uses modified silicate for emulsification.^[^ ^13^, ^15^ ^]^ Most often, hydrophobicity has been increased through silanization, e.g., with dichlorodimethylsilane, hexamethyldisilazane^[^ ^20^ ^]^ and tri‐methoxy (octadecyl)silane,^[^ ^36^, ^37^ ^]^ but some studies also performed titanium dioxide coating^[^ ^38^ ^]^ or used commercial hydrophobized colloidal silica particles.^[^ ^39^ ^]^

In recent years, there has been an increasing trend to assign an additional function to BioPE‐stabilizing particles, namely the immobilization of the biocatalysts. This increases the mass transfer at the liquid–liquid interface, which, in turn, has the potential to enable better catalytic efficiency.^[^ ^15^ ^]^ However, the direct contact of a biocatalyst with a carrier material has been shown to almost always have a direct effect on its catalytic activity.^[^ ^40^ ^]^ The extent of this effect can vary significantly depending on the material. The synthetically important lipase CalB, for example, exhibits a catalytic activity that exceeds the activity of the free enzyme by far,^[^ ^41^, ^42^ ^]^ when it is adsorbed to the commercial carrier Lewatit (Lanxess, Germany), the immobilizate being traded as Novozyme 435 (Novozymes, Denmark), while immobilization on conventional pristine silica carriers significantly reduces the activity.^[^ ^43^, ^44^ ^]^ Individual effects can be observed for different enzymes. Consequently, identifying particulate materials capable of immobilizing biocatalysts and forming w/o PE with high efficiency poses a significant challenge.

In light of the imminent widespread adoption of BioPE in synthetic biocatalysis, and considering the substantial research over several decades that has already resulted in the identification of suitable carrier materials for a wide range of biocatalysts, it would be advantageous to have a process that universally modifies these materials so that they can also stabilize emulsions. At present, however, the modification approaches described are tailored to individual particles.

In previous studies, our group has demonstrated that the wettability of macro‐sized enzyme carriers can be altered by the platinum‐catalyzed formation of polymeric silicones on the material, with hydrophilicity tuned by incorporating poly(ethylene glycol) (PEG) into the hydrophobic network.^[^ ^45^, ^46^ ^]^ The method worked with materials of different chemical composition and was compatible with the catalytic activity of both enzymes and whole cells. Therefore, we investigated here whether silicone coating could provide a method for preparing a diverse range of enzyme carriers as stabilizers for w/o PE. Inorganic and organic micro particles were included, and the effects of coating on the particle size, the size of dispersed droplets in PE, and the stability of the w/o PEs obtained were described. Finally, the benefits of BioPEs obtained with silicone‐coated particles were demonstrated using a CalB‐catalyzed transesterification as a model reaction.

## 2 Results and Discussion

### 2.1 Particle Coating

Based on the results of Scholz et al. (2013),^[^ ^45^ ^]^ silicones with different hydrophilicities were prepared by incorporating 18% (w/w) different divinyl‐terminated reactants into the hydrosilylation of SiH‐terminated poly(dimethylsiloxane) (PDMS). The hydrophilicities were characterized by determining the wettability angles against water, as described in the literature.^[^ ^18^ ^]^ However, given that biocatalyzed reactions predominantly employ buffered aqueous solutions, the wettability angles were also ascertained against solutions of potassium phosphate (KPi) at a typical concentration of 50 mmol L^−1^.

**Figure 1:**
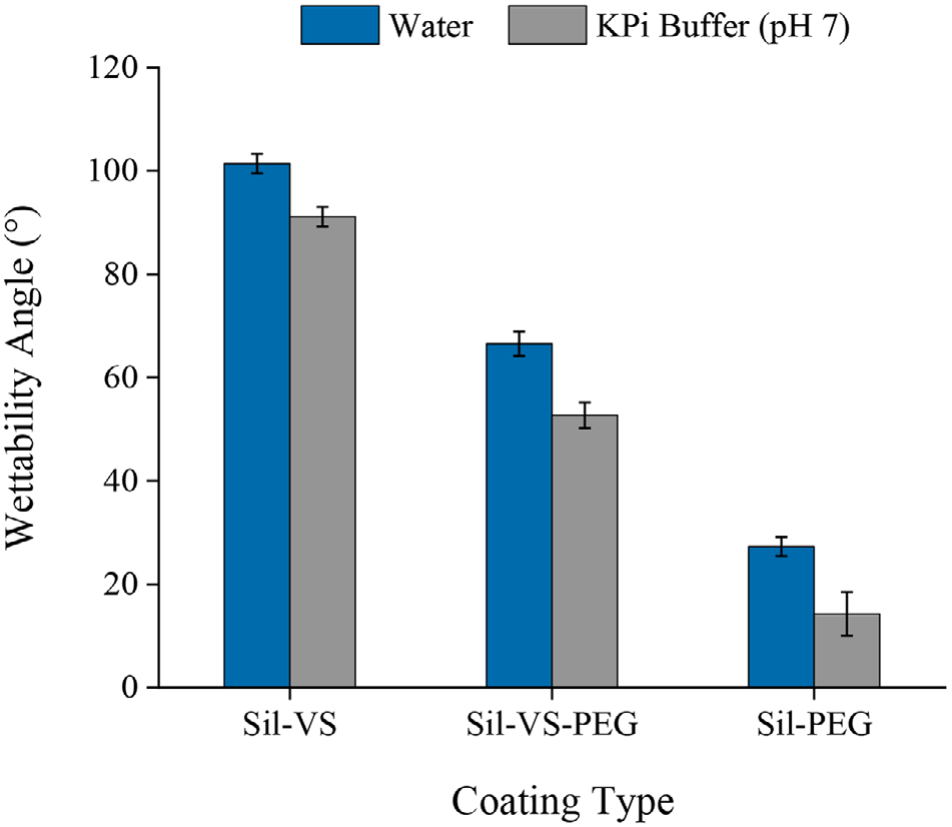
Wettability of coating materials with Milli‐Q water and 50 mmol L^−1^ KPi buffer (pH 7.0); Sil‐VS: silicone produced from PDMS and 18% (w/w) divinyl siloxane VS50; Sil‐VS‐PEG: silicone produced from PDMS, 9% (w/w) divinyl siloxane VS50 and 9% (w/w) C3‐PEG400; Sil‐PEG: silicone produced from PDMS and 18% (w/w) C3‐PEG400. Error bars represent standard deviations based on three independent experiments.

In general, the observed wettability angles were above 90° for silicones from hydrosilylation with divinyl‐terminated siloxane (VS 50), while silicones obtained with divinyl‐terminated allyl poly(ethylene glycol) 400 (C3‐PEG400) instead of VS 50 exhibited angles below 90° (**Figure** **1**; Table S1, Supporting Information) Accordingly, the materials were classified as hydrophobic silicone (Sil‐VS) and hydrophilic silicones, respectively.^[^ ^47^, ^48^ ^]^ The smallest wettability angles were found for silicones in which only C3‐PEG400 was involved in the hydrosilylation (Sil‐PEG); the incorporation of VS50 and C3‐PEG400 in equivalent masses (Sil‐VS‐PEG) was characterized by a wettability angle that was almost exactly between the wettability angles of Sil‐VS and Sil‐PEG, indicating that the angle can easily be tuned within the range set by Sil‐VS and Sil‐PEG.

For all the materials, the wettability angles were considerably smaller with buffered aqueous solution than with Milli‐Q water at the same pH (Figure 1). The effect may be related to the difference in ionic strength between the two liquids, as higher ionic strength can enhance the ionization of surface groups and increase their hydrophilicity, as shown by Virga et al. (2018).^[^ ^49^ ^]^ This observation is of particular significance in light of the previously described fact that the wettability of the material with the continuous or dispersed phase has a decisive impact on the formation of w/o or o/w PE.^[^ ^18^ ^]^ Moreover, it exerts a substantial influence on PE stability.^[^ ^22^, ^28^, ^50^ ^]^ Consequently, the reduction of the wettability angle has the potential to considerably impair the formation and stability of w/o BioPEs. Conversely, it could assist in the development and stabilization of o/w PEs, if those were the desired systems.

**Figure 2:**
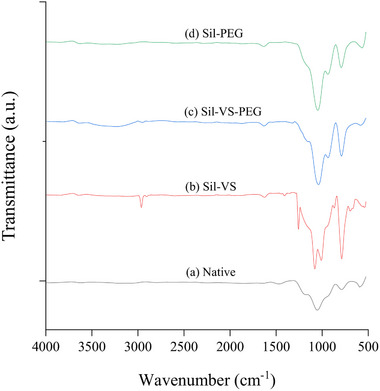
FTIR transmittance spectra of Stoeber synthesis silica particles, a) native, b) coated with Sil‐VS, c) coated with Sil‐VS‐PEG, and d) coated with Sil‐PEG.

Fourier Transform Infrared Spectroscopy (FTIR) of micro‐ and nano‐sized particles added during the hydrosilylation of PDMS showed that the silicones successfully coated inorganic and organic materials. These included Stoeber synthesis silica (**Figure** **2**), commercial silica (Sigma–Aldrich, USA), hydrophobic fumed silica‐HDK H2000 (Wacker Chemie AG, Germany), diatomaceous earth (Celite) (Merck, Germany), magnetic Fe_3_O_4,_ poly(propylene)‐based Accurel MP1001 (Membrana GmbH, Germany), and Lewatit VP OC 1600 (Lanxess, Germany), a poly(methacrylate)‐divinylbenzene copolymer (Figure S1, Supporting Information).

For example, Figure 2 shows the FTIR spectrum of native and coated Stoeber synthesis particles. Peaks typical of hydrophilic silica occur at 795,^[^ ^51^, ^52^ ^]^ 956,^[^ ^52^, ^53^ ^]^ and 1059 cm^−1^,^[^ ^54^, ^55^ ^]^ representing the Si‐O‐Si bending, Si‐O‐H stretching, and Si‐O‐Si asymmetric stretching vibrations, respectively. A peak ≈3209 cm^−1^ was attributed to free or adsorbed water. In contrast to the uncoated silica, peaks at 2964, 1631, 1412, and 1261, 1017 and 1085 cm^−1^ appeared in the Sil‐VS coated silica, corresponding to the presence of PDMS.^[^ ^56^, ^57^ ^]^ Similar peaks at 2956, 1631, 1328, and 1260 cm^−1^ were detected in the Sil‐VS‐PEG coated particles. For Sil‐PEG coated particles, a broad band ≈3330 cm^−1^ and peaks at 1060 and 800 cm^−1^ were recorded, which were in close proximity to those described in previous studies as indicative of PEG400.^[^ ^58^, ^59^, ^60^ ^]^ Again, for Sil‐VS‐PEG, similar peaks occurred, indicating the presence of PEG400 on the particle surface.

**Figure 3:**
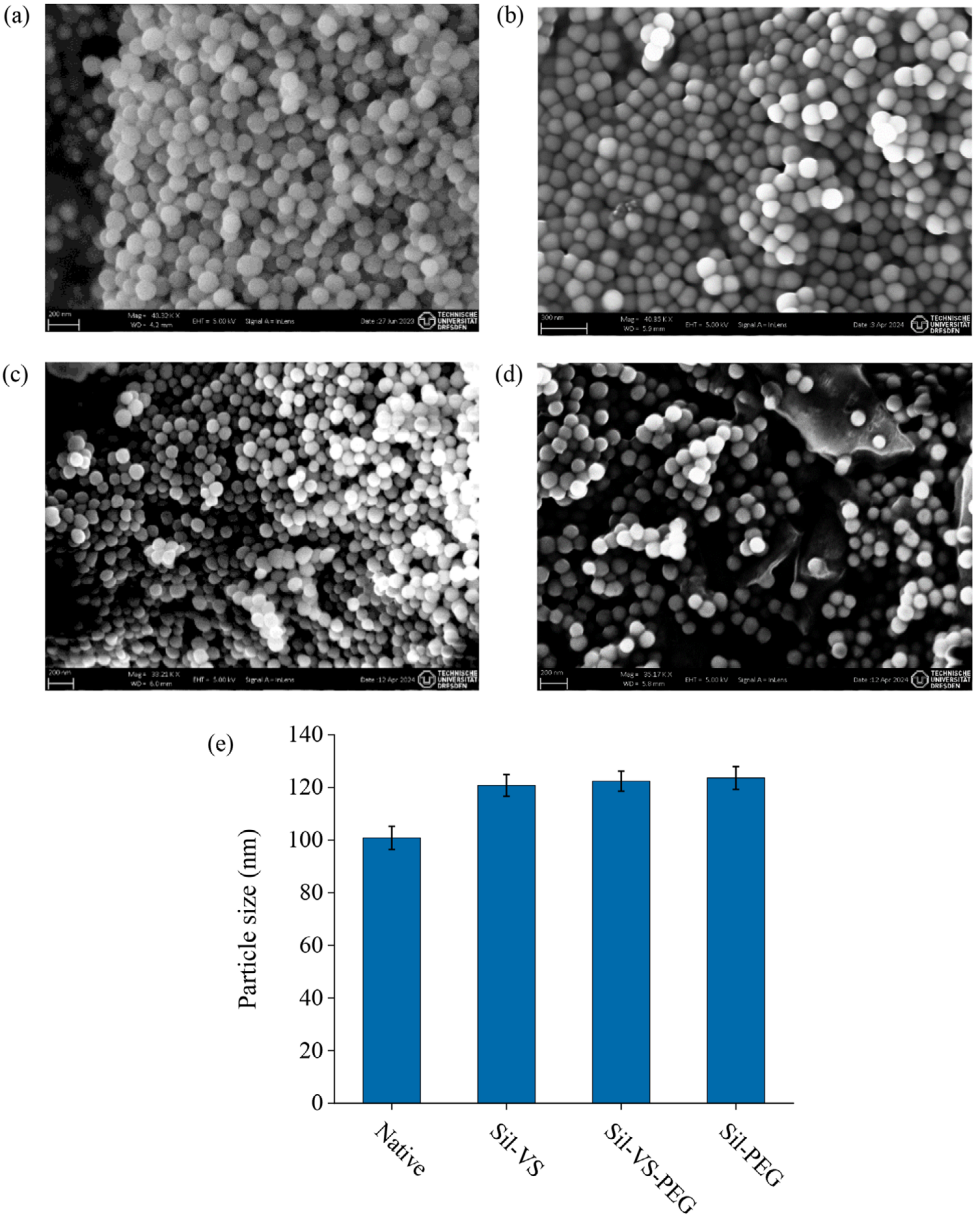
SEM of Stoeber synthesis silica particles, a) native, b) coated with Sil‐VS, c) coated with Sil‐VS‐PEG and d) coated with Sil‐PEG. e) Average size of silica particles before and after coating. Error bars represent standard deviations based on 30 particles.

Scanning electron microscopy of the Stoeber synthesis particles revealed that they retained their distinctiveness after coating, i.e., agglomeration into larger particles did not occur (**Figure** **3**). The silicone coating had no effect on the shape of the particles; however, an increase in particle size by ≈20% was observed. This increase was consistent across all types of coating and agrees with the observation of Aspinall and Khutoryanskiy,^[^ ^61^ ^]^ who observed an increase in the size of Aerosil R972 silica particles after coating with chitosan. The size increase can probably be attributed to the non‐porous nature of the particles,^[^ ^62^, ^63^ ^]^ which results in the formation of a shell‐like structure on the particle surface. Notably, a similar effect was observed after silanizing the Stoeber synthesis particles with TMODS, where a size increase of ≈10% was found (Figure S2, Supporting Information).

### 2.2 PE Stabilization

**Figure 4:**
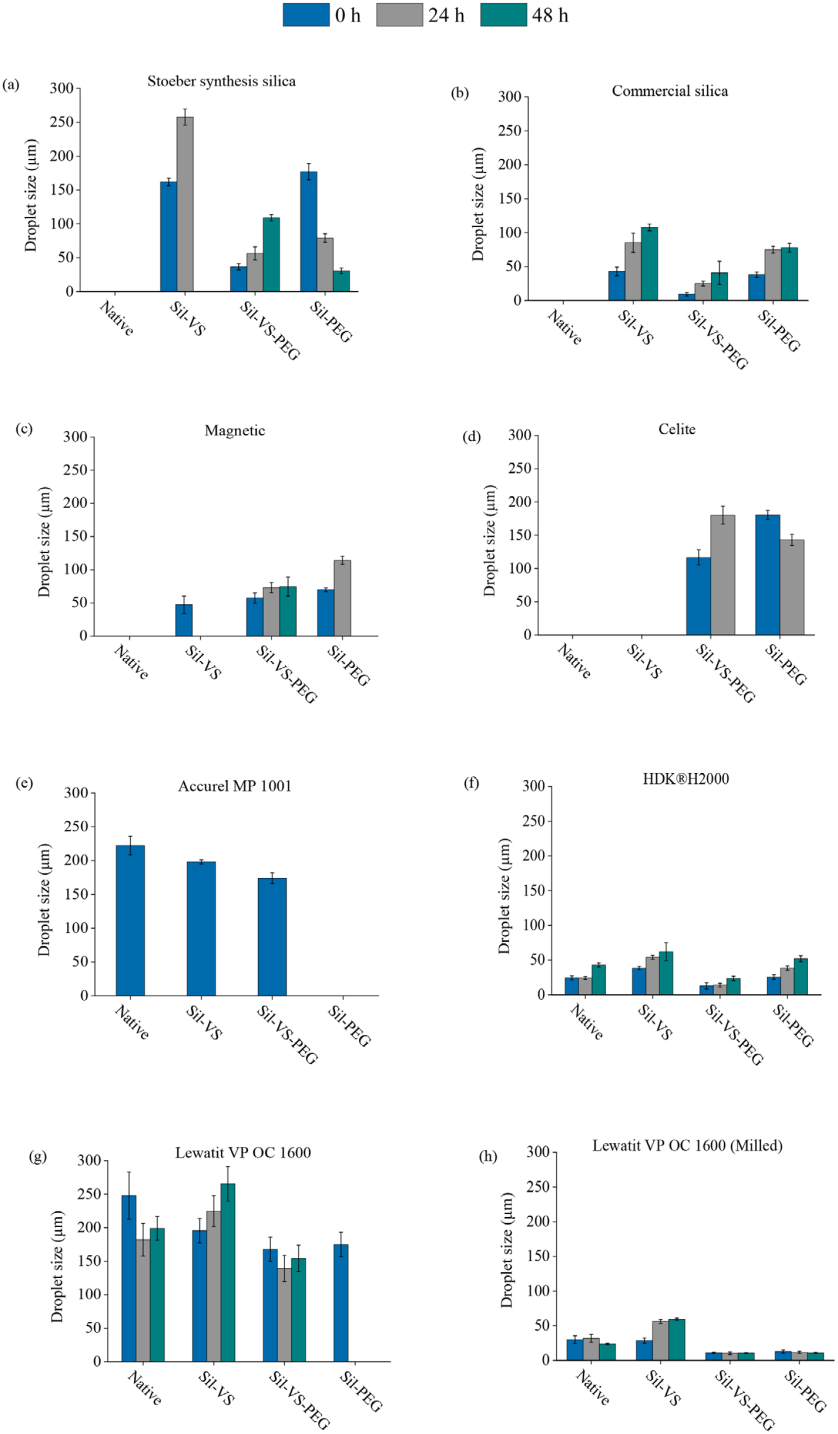
Average sizes of droplets dispersed in w/o PE directly after formation, 24 and 48 h established with a variety of native and silicone‐coated materials at a phase ratio of 33% (v_dp_/v) KPi buffer (50 mmol L^−1^, pH 7) in CPME and a particle concentration of 30 g L^−1^_dp_. a) Stoeber synthesis silica, b) commercial silica (Sigma, Germany), c) magnetic Fe_3_O_4_, d) celite, e) Accurel MP1001, f) HDKH2000 g), Lewatit VP OC 1600, and h) Lewatit VP OC 1600 after milling for 30 h at 30.1 s^−1^ with a Retsch bead mill MM300 (3 SS and 5 SS steel beads). Emulsions were stirred at 99 rpm between measurements. Error bars represent standard deviations based on three independent experiments.

Silicone‐coated particles of all studied materials were able to form w/o PEs composed of cyclopentyl methyl ether (CPME) and KPi buffer (50 mmol L^−1^, pH 7.0) as continuous oil phase and dispersed water phase, respectively (**Figure** **4**). This composition has previously been established as a suitable PE setup for biocatalysis,^[^ ^37^, ^64^, ^65^ ^]^ and was therefore utilized here.

In the case of Stoeber synthesis silica, commercial silica, celite, and magnetite particles, the silicone coating was mandatory to obtain a w/o PE. No PEs were formed with the native materials, which is consistent with the results of previous studies.^[^ ^66^, ^67^, ^68^, ^69^ ^]^ In contrast, w/o PEs were successfully formed with native Accurel MP1001, HDK H2000 and Lewatit VP‐OC 1600 particles. However, the droplets dispersed in the emulsion tended to be smaller when the materials were coated with silicone (Figure 4).

The most effective coating type for each material was contingent upon the material's initial properties. Nevertheless, Sil‐VS‐PEG frequently demonstrated the greatest efficiency in terms of the average size of the dispersed water droplets in the emulsions and their size stability over time. With the exception of Accurel MP1001 and celite, the smallest droplet sizes were observed with Sil‐VS‐PEG, and the PEs remained stable for a minimum of 48 h. Particularly small droplets (>10 µm in diameter) were obtained with Sil‐VS‐PEG‐coated commercial silica, HDK H2000, and Lewatit VP‐OC 1600 which was milled to a fine powder (Figure S3, Supporting Information). The PEs based on these particles also exhibited spherical, nearly monodisperse and tightly packed emulsion droplets. The morphologies and droplet size changes over time are illustrated in the Supporting Information (Figure S4, Supporting Information). In contrast, emulsion droplets achieved with Sil‐VS‐PEG‐coated Accurel MP 1001 were only marginally smaller than those of the uncoated material. The droplets based on Accurel MP 1001 were generally large and loosely packed, and the emulsions rapidly disintegrated (Figure S4, Supporting Information). The observations are likely attributable to the sizes of the stabilizing particles, as numerous studies have established a direct proportionality between particle size and emulsion droplet size, along with an inverse proportionality to emulsion stability.^[^ ^70^, ^71^, ^72^ ^]^ Commercial silica,^[^ ^73^ ^]^ HDK H2000,^[^ ^74^, ^75^ ^]^ and milled Lewatit particles (Figure S3, Supporting Information), show very small diameters, whereas according to the manufacturers’ instruction leaflet, Accurel MP 1001 particles have a size range of 400–1000 µm. Accordingly, large droplet sizes (>150 µm in diameter) and low PE stability were also observed with the commercial (unmilled) Lewatit VP‐OC 1600 particles, which have large diameters ranging from 320 to 450 µm.^[^ ^76^ ^]^ Finally, it is noteworthy that for all silica‐based particles, both droplet size and PE stability were significantly better after particle coating with Sil‐VS‐PEG compared to silanization with TMODS (Figure S5, Supporting Information). This suggests that, within our experimental setup, Sil‐VS‐PEG‐coated silica particles manifest a surface hydrophilicity that is better suitable for w/o PE than TMODS‐coated particles.

The majority of the Sil‐VS‐ and Sil‐PEG‐coated particles also demonstrated the capacity to form PEs, though the droplet sizes and the PE stability were predominantly less optimal compared to the Sil‐VS‐PEG‐coated materials (Figure S4, Supporting Information). This observation is particularly salient in the context of Sil‐PEG, given the distinct hydrophilicity of this material, as evidenced by the low wettability angles observed (Figure 1). In fact, stabilization of o/w PE rather than w/o PE could have been expected, since spherical particles with wettability angles of 15° < θ < 90° are deemed most suitable for stabilization of o/w emulsions.^[^ ^18^, ^20^, ^77^ ^]^ However, the material demonstrated the capacity to elevate the surface hydrophobicity of Stoeber synthesis silicate, commercial silica, and celite to a level sufficient to enable PE formation that was not possible with the native particles. Conversely, Sil‐PEG demonstrated an inability to induce PE formation in the presence of Accurel MP 1001, despite the capacity of native Accurel MP 1001 to do so. Similarly, the most hydrophobic coating, Sil‐VS, did not enable PE formation with celite. These observations suggest a potential incompatibility between very hydrophobic particles and very hydrophilic silicone coatings, and vice versa, although both Sil‐PEG and Sil‐VS were consistently found to be present on the surfaces of Accurel MP 1001 or celite, respectively (Figures S1c,d, Supporting Information).

In general, the utilization of SIL‐VS‐coated particles resulted in the induction of PE exhibiting enhanced stability in comparison to PE derived from Sil‐PEG‐coated particles. However, when evaluated in conjunction with Sil‐VS‐PEG‐coated particles, the emulsion droplets exhibited significantly larger dimensions for both materials. In light of the augmented amphiphilic character of the Sil‐VS‐PEG coating, this finding aligns with the observations reported by Briggs et al.^[^ ^78^ ^]^ These researchers noted that amphiphilic multi‐walled carbon nanotubes led to the induction of PE with reduced droplet sizes and enhanced stability in comparison to their hydrophobic or hydrophilic counterparts.

The stability behavior of the emulsions obtained with Sil‐PEG‐coated Stoeber synthesis silicate and celite particles is rather peculiar. Contrary to the usual trend, a decrease in the average droplet diameter occurred over time. However, we suspect that this phenomenon is not directly related to the coating type, but rather is due to a statistical effect. Emulsions stabilized by Sil‐PEG‐coated Stoeber synthesis particles exhibited an exceptionally polydisperse droplet distribution, which decreased significantly over time. Initially, droplets with a large diameter were clearly in the majority (Figure S4, Supporting Information). Given the reduced stability of such large droplets in comparison to their smaller counterparts, it can be deduced that they dissolved rapidly, while the diameter of the smaller droplets remained constant or increased only gradually. Consequently, the relative scarcity of large droplets in the system led to the observation of a reduced mean droplet size, resulting in an apparent decrease over time. This assumption is further substantiated by the observation that the average droplet size in PEs stabilized by Sil‐PEG‐coated celite particles was notably smaller after 24 h than at the beginning, while the emulsion was completely dissolved after 48 h. The polydispersity of this emulsion was initially significantly lower than in the emulsion stabilized by Sil‐PEG‐Stoeber synthesis silicate. However, the initial proportion of very large droplets also predominated (Figure S4, Supporting Information).

Additionally, emulsions stabilized with Sil‐VS‐PEG‐coated particles had smaller droplet sizes than those stabilized with native or other coated particles. Considering the influence of surface properties on droplet size, this can be explained by a stronger adsorption of amphiphilic Sil‐VS‐PEG‐coated particles to the oil‐water interface compared to more hydrophilic Sil‐PEG‐coated particles and more hydrophobic Sil‐VS‐coated particles. Consequently, the tendency to coalesce is reduced, and smaller droplets are produced.^[^ ^78^, ^79^ ^]^ With a more hydrophilic or hydrophobic surface, the particles tend to remain in the water or organic phase due to stronger wetting by one of the phases, leading to poorer interfacial coverage, requiring more particles to achieve complete coverage, higher stability, and smaller droplet size.

In summary, our observations indicate that, with regard to PE formation, the surface characteristics of the silicone coating predominate over the initial properties of the particles, rather than modifying them. PEs stabilized with predominantly hydrophobic or hydrophilic coated particles are more prone to destabilization than those with amphiphilic coating. This phenomenon can be attributed to the propensity of hydrophobic or hydrophilic particles to become predominantly wetted by one of the liquids, which renders them vulnerable to desorption from the water‐oil interface.^[^ ^80^, ^81^ ^]^

### 2.3 Performance in BioPE

The practical benefit of particle coating with silicone for the establishment of BioPE was evaluated using CalB‐mediated biocatalysis, which is currently the best studied and most extensively optimized in PE. Based on the observations from the preceding experiments, Sil‐VS‐PEG was selected for coating. In order to facilitate a direct comparison of BioPE generated with Sil‐VS‐PEG coated particles and CalB in the dispersed phase with systems known from literature, silicate particles were used to stabilize the PE, and silanization with TMODS was included in the investigation. Given the observation that both Sil‐VC‐PEG‐coated commercial silica and Stoeber synthesis silica particles exhibited analogous properties with regard to the stabilization of PE, yet the droplet size distribution with commercial silica was more favorable (Figure 4a,b), commercial silica was utilized in the experiments.

**Figure 5:**
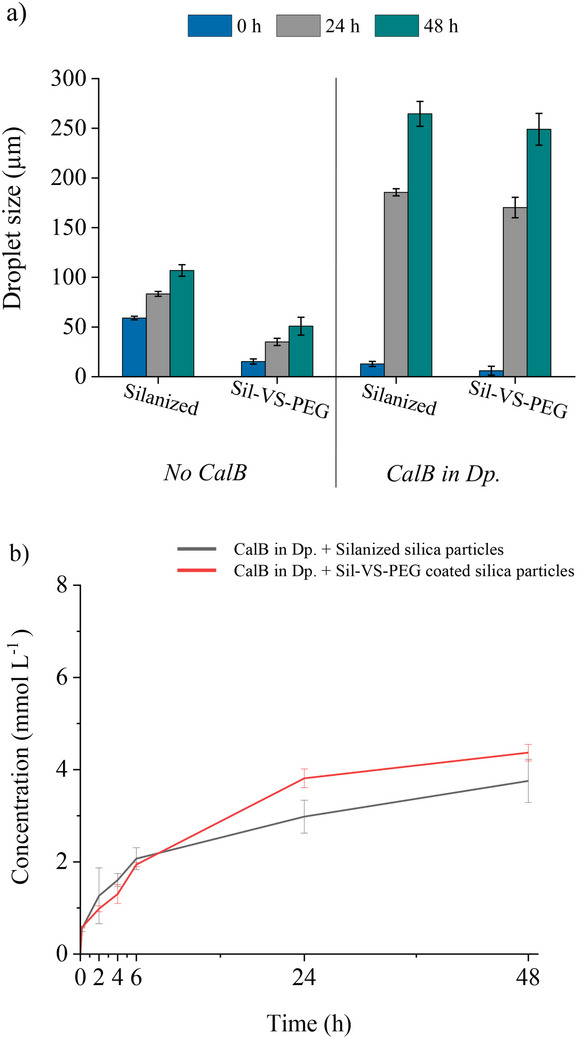
Stability and catalytic activity of w/o BioPE established with 30 g L_dp_^−1^ of TMODS‐silanized or Sil‐VS‐PEG coated commercial silica at a phase ratio of 33% (v_dp_/v) KPi buffer (50 mmol L^−1^, pH 7) in CPME, without enzyme, and with 1.9 g L_dp_^−1^ lipase CalB, respectively. a) Droplet sizes directly after PE formation, after 24 h and after 48 h; b) Concentration of the product 1‐phenylethyl butyrate from the transesterification of 1‐phenylethanol with vinyl butyrate. Emulsions were stirred at 99 rpm between measurements; error bars represent standard deviations based on three independent experiments.

In the experimental setting, the addition of the lipase to the dispersed phase (dp) of the BioPE yielded substantial variations (**Figure** **5**a). Irrespective of the coating method employed on the particles, whether with silicone or TMODS, the initial size of the emulsion droplets was reduced by approximately twofold when CalB was present, compared to the enzyme‐free emulsion. Concurrently, the stability of the BioPE was significantly compromised, as evidenced by the considerable increase in droplet size over time, accompanied by a decrease in catalytic activity (Figure 5b).

These observations were consistent with the findings from one of our previous studies on the effect of enzyme addition to w/o PE,^[^ ^65^ ^]^ where we hypothesized that globular proteins, such as CalB, move to the emulsion interface and then act as emulsifiers themselves. However, due to their lack of typical emulsifying properties,^[^ ^82^ ^]^ they are unable to stabilize the PEs. Consequently, their interaction with the interface leads to the destabilization of PEs,^[^ ^65^ ^]^ resulting in a reduced interfacial area due to increased droplet sizes. This, in turn, most probably lowers the catalytic activity because of a reduced substrate‐enzyme contact.

**Figure 6:**
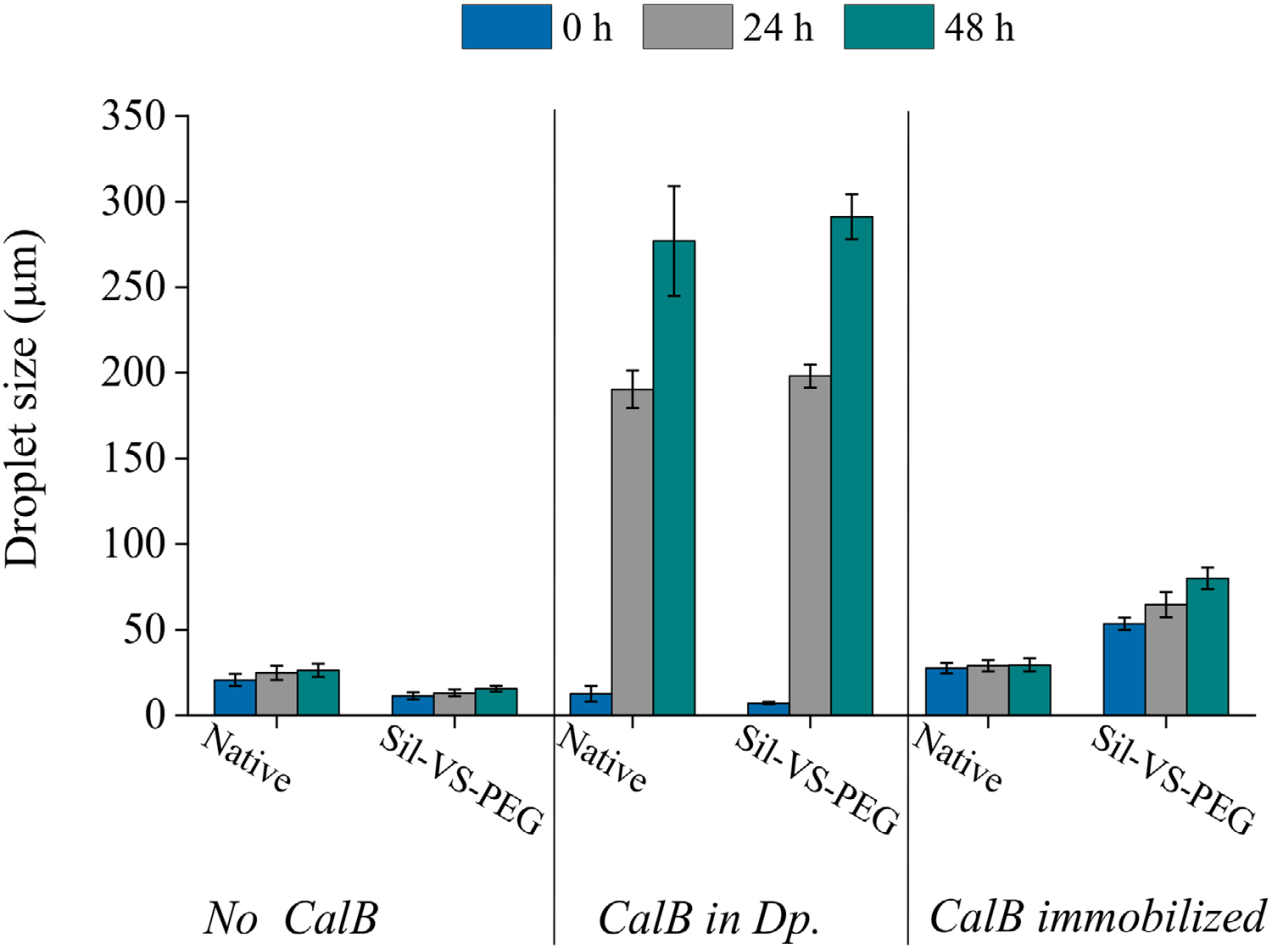
Average droplet sizes in w/o PE established with 30 g L^−1^_dp_ of uncoated and Sil‐VS‐PEG‐coated milled Lewatit VP OC 1600 particles, respectively, at a phase ratio of 33% (v_dp_/v) KPi buffer (50 mmol L^−1^, pH 7) in CPME directly after formation, after 24 h and after 48 h. Emulsions contained no enzyme, 1.9 g L_dp_^−1^ enzyme in the dispersed phase, or 0.06 g g _np_^−1^ enzyme on the particles, and were stirred at 99 rpm between measurements. Error bars represent standard deviations based on three independent experiments.

Subsequent experiments demonstrated that the destabilizing effect of enzymes on PEs can be circumvented or, at the least, mitigated by their location on the particles' surface (**Figure** **6**), thereby demonstrating another advantage of the immobilization of biocatalysts in BioPE. The size increase of the emulsion droplets in the PE over time was considerably reduced when CalB was adsorbed to the stabilizing particles instead of being suspended in the dp. The initial droplet size was only slightly increased. The subsequent coating of Sil‐VS‐PEG on the particles led to a two‐fold increase of the initial droplet size and a further enhancement of the time‐dependent increase in droplet size. Notwithstanding, the PE stability remained significantly superior to that of the PE with CalB suspended in the dp.

Given the incompatibility of lipase with pristine silicate (Table S2, Supporting Information), which has been demonstrated to result from rapid enzyme denaturation and compromised activity,^[^ ^43^ ^]^ we employed Lewatit VP OC 1600 (Lewatit) as a PE‐stabilizing material for these experiments for the first time. As described in the introduction, Lewatit is a well‐established carrier material for lipases, particularly CalB. In light of the observations outlined in the previous section, the commercial Lewatit was ground to a fine powder before being used to immobilize the biocatalyst and stabilize the PE. On the milled Lewatit, a protein loading of 0.06 g_protein_ g_nanoparticle_^−1^ (g_p_ g_np_^−1^) and a hydrolytic activity for p‐nitrophenyl acetate of 0.43 U mg_np_^−1^ was achieved.

**Figure 7:**
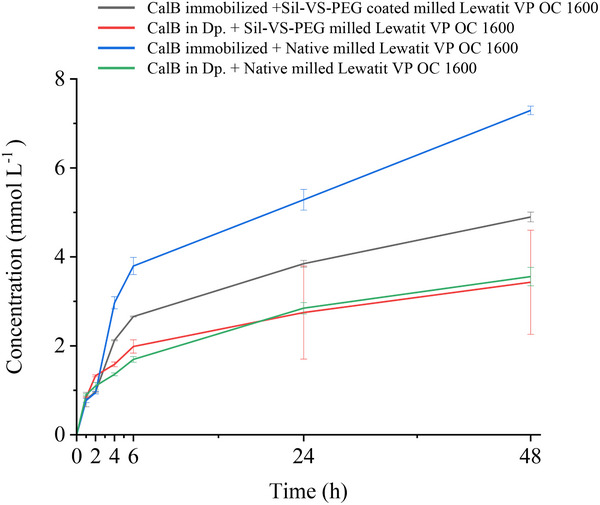
Concentration of the product 1‐phenylethyl butyrate from the transesterification of 1‐phenylethanol with vinyl butyrate in w/o PE established with 30 g L_dp_^−1^ of uncoated and Sil‐VS‐PEG‐coated milled Lewatit VP OC 1600 particles, respectively, at a phase ratio of 33% (v_dp_/v) KPi buffer (50 mmol L^−1^, pH 7) in CPME directly after formation of the emulsion, after 24 h and after 48 h. Emulsions contained 1.8 g L_dp_^−1^ enzyme in the dispersed phase, or 0.06 g_p_ g_np_^−1^ enzyme on the particles, and were stirred at 99 rpm between measurements. Error bars represent standard deviations based on three independent experiments.

In Lewatit‐stabilized BioPE, CalB showed transesterification activity in both configurations: dissolved in the dp and immobilized on the particles (**Figure** **7**). However, the enzyme immobilized on the particles yielded more product. The uncoated particles performed slightly better than the Sil‐VS‐PEG‐coated particles, indicating that the coating had only a minor effect on the activity, presumably by limiting mass transfer. At the beginning of the reaction, the productivity of the BioPEs with dissolved enzymes was slightly higher, probably due to the smaller initial droplet size. Correspondingly, the productivity decreased rapidly as the droplet size increased during the reaction (Figure 6).

## 3 Conclusion

Silicone coatings proved to be a straightforward solution for adapting microparticles with different chemical compositions and surface functionalities for the stabilization of PEs, which distinguishes the method from other surface modifications. For example, silanization requires surfaces saturated with hydroxyl groups^[^ ^83^ ^]^ and is most compatible with silica and glass surfaces,^[^ ^84^ ^]^ whereas modification of organic polymers requires individual treatment.^[^ ^85^ ^]^ The emulsifying properties of the silicone‐coated particles presented here are dependent on the silicone composition rather than the inherent properties of the native particles, whereby the effect of the coating is foreseeable for a wide variety of materials. At the same time, the hydrophobicity of the silicone, and consequently the wettability of the particles, can be easily adjusted from a hydrophobic to a hydrophilic state, providing a high degree of flexibility according to the requirements of the emulsion system. The performance of silicone‐coated particles in stabilizing PE is comparable to, or even superior to, established native or modified particles.

The coating also conforms to the enzymes bound to the particle surface prior to coating, with only a slight reduction in catalytic activity. As a result, the silicone coating has the potential to expand the range of micro particles that can be used in the production of w/o BioPE, along with enhancing the applicability of PE for biocatalytic production. Moreover, well‐established enzyme carriers can also be used, as demonstrated with a commercial Lewatit carrier, facilitating the transfer of the catalytic reaction to the emulsion interface and offering increased productivity. Furthermore, issues with emulsion stability, which arise from the utilization of dissolved enzymes in the dispersed phase, are mitigated.

In summary, silicone coating provides a straightforward, versatile, and tunable method for the preparation of particles that provide suitable properties for both the stabilization of BioPE and the immobilization of active biocatalysts. Consequently, it possesses the capacity to exert a favorable influence on the transfer of BioPE into practical applications. It can also be expected that the silicone coating can be further expanded into o/w emulsions, considering that the method can be conveniently adjusted to suit specific PE setups and catalyst properties.

## 4 Experimental Section

### Chemicals

Unless otherwise indicated, all chemicals and solvents were obtained from commercial suppliers, including Carl Roth (Germany), Sigma‐Aldrich (USA), Thermo Fisher Scientific (Germany), and VWR (USA), with the highest available purity and used as received. Detailed information, including the CAS registry number, is given in Table S3 (Supporting Information). Divinyl‐terminated siloxane (D‐siloxane) was provided by Evonik Industries AG, (Germany).

### Particle Acquisition

Silica (SiO_2_) nanoparticles (10–20 nm) were purchased from Sigma‐Aldrich (USA); Accurel MP1001 (400–1000 µm) was a kind gift from Membrana GmbH (Germany); Celite was purchased from Merck (Germany); HDKH2000 (12–18 nm) was obtained from Wacker Chemie AG (Germany) and, Lewatit VP OC 1600 (0.32–0.45 mm) was obtained from Lanxess (Germany).

### Synthesis of Magnetic Particles

Magnetic Fe_3_O_4_ particles were synthesized as outlined by Petcharoen and Sirivat (2012).^[^ ^86^ ^]^ Briefly, ferrous chloride tetrahydrate (1.5 g) and ferric chloride (3.0 g) were dissolved in deionized water (100 mL) at 45 °C under nitrogen gas flow, followed by the addition of ammonium hydroxide (10 mL, 25% w/w). The obtained magnetic particles were thoroughly washed with deionized water and dried in a Martin Christ Alpha 1–4 LSC basic lyophilizer (Germany) for 24 h.

### Stoeber Synthesis of Silica (SiO_2_) Particles

The SiO_2_ particles were synthesized following a modified Stoeber process.^[^ ^87^, ^88^, ^89^ ^]^ A mixture consisting of methanol (38.85 mL), ammonia (6.12 mL, 25% w/v) and deionized water (3.15 mL) was briefly stirred using a Heidolph MR 3001 magnetic stirrer (Germany) at 40 °C and 500 rpm. Tetraethyl orthosilicate (1.9 mL) was added while stirring vigorously, and the speed was reduced to 300 rpm for the next 2 h. The mixture was then centrifuged, and the particles obtained were washed three times with water. The size distribution of the particles was assessed using a Carl Zeiss Supra 40VP scanning electron microscope (SEM) (Germany) by loading dry sample powder onto graphite disks supported by aluminium stubs. Measurements were performed at an accelerating voltage of 5 kV. Fourier Transform Infrared (FTIR) spectra were recorded using a Thermo Scientific–Nicolet 8700 with an attached Thermo Scientific Smart iTR in Attenuated Total Reflectance (ATR) mode FTIR spectrometer (USA) in the range of 450–4000 cm^−1^. Powder samples were used for the measurement.

### Milling Lewatit VP OC 1600

The Lewatit VP OC particles (0.32–0.45 mm) were oven‐dried for 3 days at 50 °C and then milled for 30 h at a frequency of 30.1 s^−1^ with a Retsch mixer mill MM300 (Germany) using a mixture of 3 SS and 5 SS steel beads. The size of the ground particles was determined using an Anton Paar Litesizer 500 dynamic light scattering (DLS) instrument (Austria) and the Kalliope Anton Paar software. The particles were dispersed in ethanol in a 2.5 mL macro PS cuvette (12.5 ⋅ 12.5 ⋅ 45 mm) and subjected to 60 runs per measurement at a temperature of 25 °C and a scattering angle of 175° (Figure S3, Supporting Information).

### Silicone Coatings

Silicone coatings were prepared using a modified version of Findeisen (2013, 2014).^[^ ^46^, ^90^ ^]^ For amphiphilic and hydrophilic coatings, C3‐PEG400 was synthesized as described by Findeisen (2013).^[^ ^46^ ^]^ Briefly, PEG400 (5 mmol) and solid NaOH (0.1 mol) were added to a round‐bottomed flask containing 100 mL of toluene. The contents of the flask were stirred at 1000 rpm for 20 h at room temperature using a Heidolph MR 3001 magnetic stirrer (Germany). Then, allyl chloride (0.1 mol) was added to the flask, and the mixture was decanted into another flask, and the supernatant was removed using a Rotavapor R‐100 rotary evaporator (Germany). Specifics of the different coatings are given in **Table** **1**.

**Table 1:** Composition of silicone coatings.

Coating	Component	Quantity
Hydrophobic silicone (Sil‐VS)	D‐Siloxane	20 mg
	Polymer VS 50	91 mg
	Isopropanol	9 mL
	Hexane	1 mL
Amphiphilic silicone (Sil‐VS‐PEG)	D‐Siloxane	20 mg
	Polymer VS 50	45.5 mg
	C3‐PEG 400	45.5 mg
	Isopropanol	9 mL
	Hexane	1 mL
Hydrophilic silicone (Sil‐PEG)	D‐Siloxane	20 mg
	C3‐PEG 400	91 mg
	Isopropanol	9 mL
	Hexane	1 mL

For particle coating, the components for the desired coating type were mixed as shown in Table 1 and incubated at *4* *°C* and 80 rpm. In addition, particles (500 mg) were added to a solution of isopropanol and hexane (5 mL, 10:1 ratio), and the mixture was allowed to stand overnight at 4 °C and 80 rpm. The two mixtures were then combined, Karstedt's catalyst (5 µL) was added, and the mixture was incubated for 3 h at 4 °C and 500 rpm. Finally, the mixture was dried in an oven at 50 °C overnight to complete polymerization.

For wettability measurements, glass slides were prepared by immersion in hydrosilylation solutions for 3 h and then dried at 50 °C overnight. Wettability was determined using a DataPhysics Instruments OCA 15ec optical angle goniometer (Germany) with an attached light source and camera. Static contact angles were measured using the sessile drop technique, in which a drop of deionized water (1 µL) or KPi buffer (50 mmol L^−1^, pH 7.0) was applied to the coated slide using a microsyringe. The image of the drop was recorded, and the contact angles were measured using the SCA20 software.

### Silanization

Silanized particles were prepared by stirring a mixture of particles (10 g) with pure, denatured ethanol (930 mL), trimethoxy(octadecyl)silane (TMODS) (40 mL), and ammonia (30 mL, 25% w/v) at 60 °C and 600 rpm overnight. The resulting particles were washed three times with ethanol and dried at 50 °C in an oven overnight. The modified particles were characterized using FTIR and SEM in a similar manner as explained earlier (Stoeber Synthesis of Silica (SiO_2_) Particles).

### PE Setup and Characterization

A water‐in‐oil (w/o) PE (25 mL) was formulated using a KPi buffer (50 mmol L^−1^ pH 7) as the dispersed phase (dp) and CPME as the continuous phase (cp). The dp content was 33% (v/v), the cp composition was 67% (v/v), and the particle content was 3% w/v of the dp. The particles were introduced into the cp and dispersed using a vortex mixer for 2 mins, followed by sonication for 10 min. IKA T18 digital Ultra‐Turrax (Germany) was then used at 17 500 rpm for 20 s to achieve the final dispersion. The dp was then added, and the Ultra‐Turrax was used at 17 500 rpm for 2 min to develop a PE. For stability measurements, the emulsified droplets were imaged for 0, 24, and 48 h, using a camera attached to a Nikon eclipse Ts2 inverted electron microscope (Japan) at 4×, 10×, and 20× magnification. A maximum of 500 and a minimum of 50 droplets per PE setup were analyzed using the Smart Online Particle Analysis Technology (SOPAT) image analysis software (SOPAT, Germany). The PE was incubated at room temperature and 99 rpm on a Labinco LD 79 rotator (Netherlands) throughout the experiment. All experiments were carried out in independent triplicate.

### Provision of CalB

The production of wild type CalB (lipase B from *Candida antarctica*) was achieved through the use of the Nico 21 (DE3) *E. coli* expression system, as described in Steinhagen et al.^[^ ^42^ ^]^ The purity of the enzyme was confirmed by performing SDS‐PAGE (Figure S6, Supporting Information), while the concentration of the enzyme was determined using a Thermo Scientific NanoDrop ND‐1000 UV/Vis Spectrophotometer (USA). The enzyme activity for purified CalB was measured on a spectrophotometer (DR 3900, Hach–Lange GmbH, Germany) by determining the conversion rate of p‐nitrophenyl acetate (pNPA) to p‐nitrophenol (pNP). For this, pNPA (100 mmol L^−1^ in methanol) was mixed with Tris‐HCl buffer (50 mmol L^−1^, pH 8) in a 3 mL cuvette. The reaction was initiated by the addition of the enzyme solution, and the formation of pNP was quantified by the increase in absorbance at 405 nm. Approximately 40 mg of pure CalB with a specific activity of 18 ± 10.1 U mg^−1^, based on pNPA hydrolysis, was obtained from a 1‐L batch culture. Volumetric activity was determined according to Equation (1), and specific activity according to Equation (2).

(1) $$A_{\mathrm{vol}}=\frac{\Delta E\;min^{-1}\times V_{total}}{d\times V_{enzyme}\times \varepsilon }$$

(2) $$A_{spec.}=\frac{A_{vol.}}{c}$$

A_vol_ : volumetric activity in units / mL_enzyme solution_
*Δ*E min ^−1^ : extinction change over minute at λ 405 nm min^−1^ over the linear range
V_total_ : total volume of the reaction mixture (mL)
d : layer thickness of cuvette (cm)
V_enzyme_ : volume of the enzyme solution (mL)
ε : molar extinction coefficient 𝑝NP (18 000 M^−^¹ cm^−^¹)
A_spec_ : specific activity of the enzyme
*c* : protein concentration (mg mL^−1^)

The purified CalB was lyophilized on an Alpha 1–4 LSCbasic, (Christ, Germany) and stored at −80 °C until further use.

### Immobilization of CalB

Milled Lewatit VP OC 1600 particles (0.25 g) were dispersed in pure, denatured ethanol and then incubated for a period of 30 min at room temperature and 300 rpm on a magnetic stirrer. Following this, the particles were thoroughly washed with KPi buffer (500 mmol L^−1^, pH 7) and allowed to dry in air for a period of 3 h. The particles were then immersed in a solution of CalB (4 mg mL^−1^) in a total volume of 4 mL, and incubated for 24 h at 4 °C and 36 rpm on a rotating mixer. At the conclusion of the incubation period, a sample of the medium and the particles was collected for the determination of the enzyme loading on the particles using the Thermo Fisher Scientific Pierce BCA kit (USA). The loaded particles were dried over silica gel at 4 °C for five days, followed by the silicone coating procedure described in Experimental Section under the heading “Silicone coatings,” omitting the overnight particle wetting step. Upon completion of the process, an additional sample of the particles was taken to assess CalB content in the coated particles via BCA assay. The calibration curve for the BCA assay was established using CalB as the standard. All measurements were carried out in triplicate. The (apparent) specific activity of the immobilized enzyme was calculated using the pNPA hydrolysis assay (Provision of CalB) and replacing V_enzyme_ in Formula 1 by M_particles_ (mass of particle with immobilized enzyme).

### Conduction of BioPE

The catalytic performance of CalB in PEs was evaluated for the transesterification of 1‐phenylethanol with vinyl butyrate, resulting in the formation of 1‐phenylethyl butyrate. The PEs were configured as outlined in the Experimental Section under the heading “PE Setup and Characterization,” either with the addition of CalB in KPi buffer before formation of PE or with the substitution of coated particles with CalB‐immobilized and coated particles. Subsequently, vinyl butyrate (581 mmol L^−1^) and 1‐phenylethanol (82 mmol L^−1^), were introduced into the system, and samples were collected over a period of 48 h by centrifuging 700 µL of the emulsion and extracting the top organic phase The samples were analyzed for 1‐phenylethyl butyrate using a AOC‐20i gas chromatographer (Shimadzu Corp., Japan). All measurements were carried out in triplicates. Additionally, droplet sizes were measured at 0, 24, and 48 h, as described in Experimental Section under the heading “PE Setup and Characterization.”

## Conflict of Interest

The authors declare no conflict of interest.

## Supporting information



Supporting Information

## Data Availability

Data will be available from the corresponding author upon reasonable request.
