# Supplementary material for: Expanding the Particle Range for Biocatalytically Active Pickering Emulsions with Silicone Coatings
Source: Adv Mater. 2025 Jul 22;37(41):e08738. doi: 10.1002/adma.202508738 (PMC12531736; doi:10.1002/adma.202508738)
Supplement: Supplementary file 1 — Supporting Information [file ADMA-37-e08738-s001.pdf]

ADVANCED MATERIALS

Supporting Information

for *Adv. Mater.*, DOI 10.1002/adma.202508738

Expanding the Particle Range for Biocatalytically Active Pickering Emulsions with Silicone Coatings

*Sara Fatima Bhutta, Christoph Plikat and Marion B. Ansorge-Schumacher**

Supporting Information

Expanding the particle scope for biocatalytically active Pickering emulsions with silicone coatings

Sara Fatima Bhutta , Christoph Plikat, Marion B. Ansorge-Schumacher[1]

[1]Technische Universität Dresden, Institute for Microbiology, Chair of Molecular Biotechnology, Zellescher Weg 20b, 01217 Dresden, Germany

[*]Correspondence should be addressed to: marion.ansorge@tu-dresden.de

Table SI-1: Wettability angle images of hydrophobic (Sil-VS)**,** amphiphilic (Sil-VS-PEG), and hydrophilic (Sil-PEG) coatings with Milli-Q water and 50 mmol L^{-1} KPi buffer (pH 7.0), respectively. The coatings were spread on clean glass slides, and the average of triplicate measurements was recorded.

**Coating type**	**Wettability angle with water**	**Wettability angle with KPi buffer (pH 7.0)**
Sil-VS	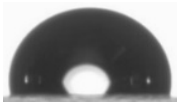 101.4°	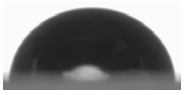 91.1°
Sil-VS-PEG	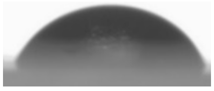 66.5°	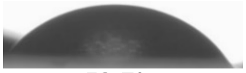 52.7°
Sil-PEG	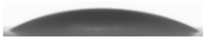 27.3°	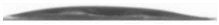 14.3°

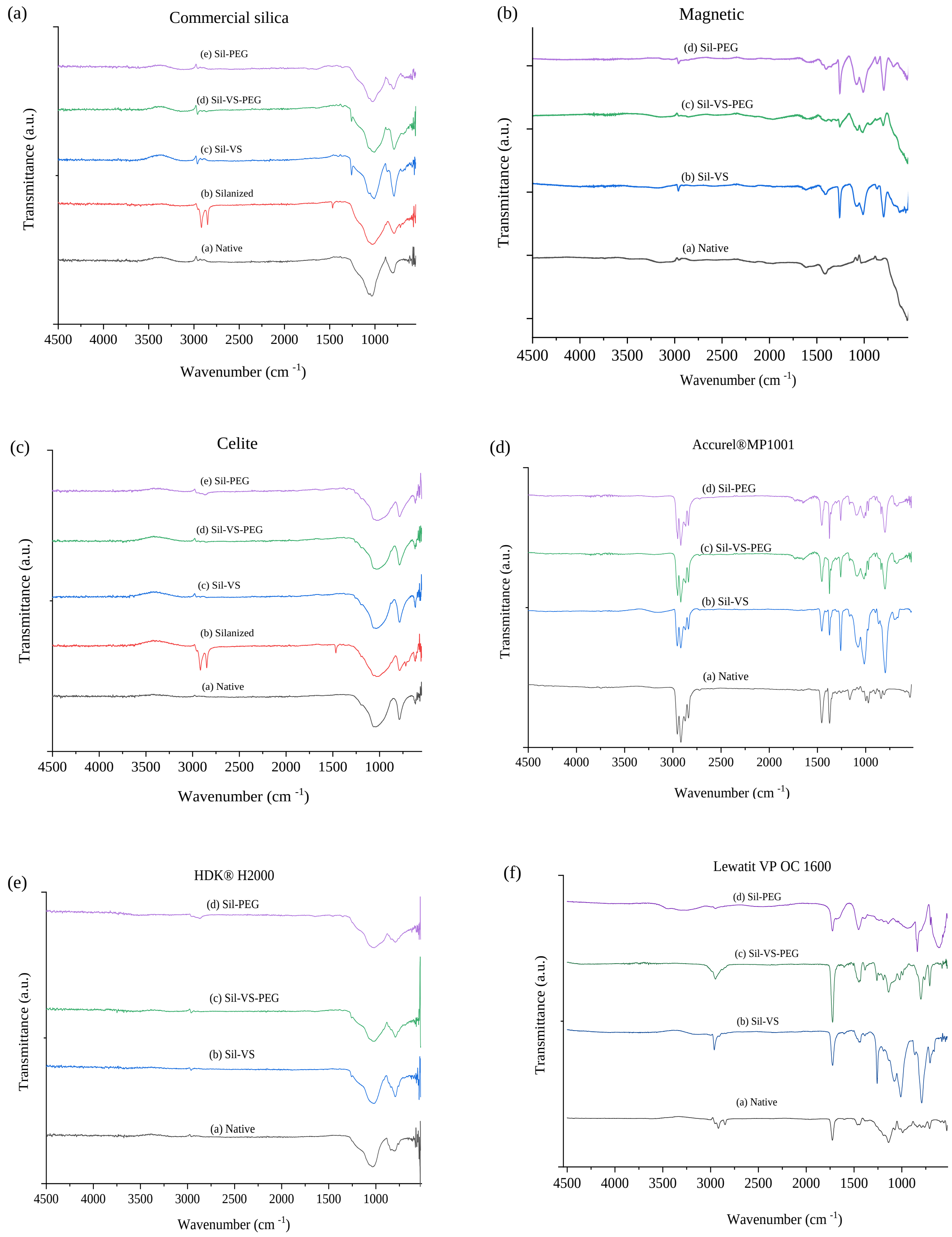

Figure SI-1: Figure SI-1: FTIR spectra of particles without coating, native particles, silanized particles, and particles coated with Sil-VS, Sil-VS-PEG and Sil-PEG. (a) Commercial silica, (b) Magnetic, (c) Celite, (d) Accurel MP 1001, (e) HDK ® H2000, and (f) Lewatit VP OC 1600.

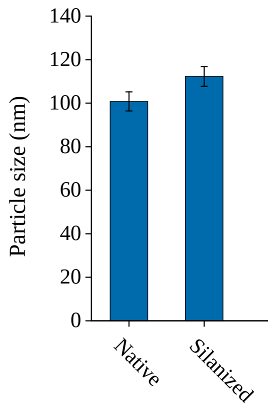

Figure SI-2: Average size of silanized Stoeber synthesis silica particles analyzed with scanning electron spectroscopy (SEM).

(a)

(b)

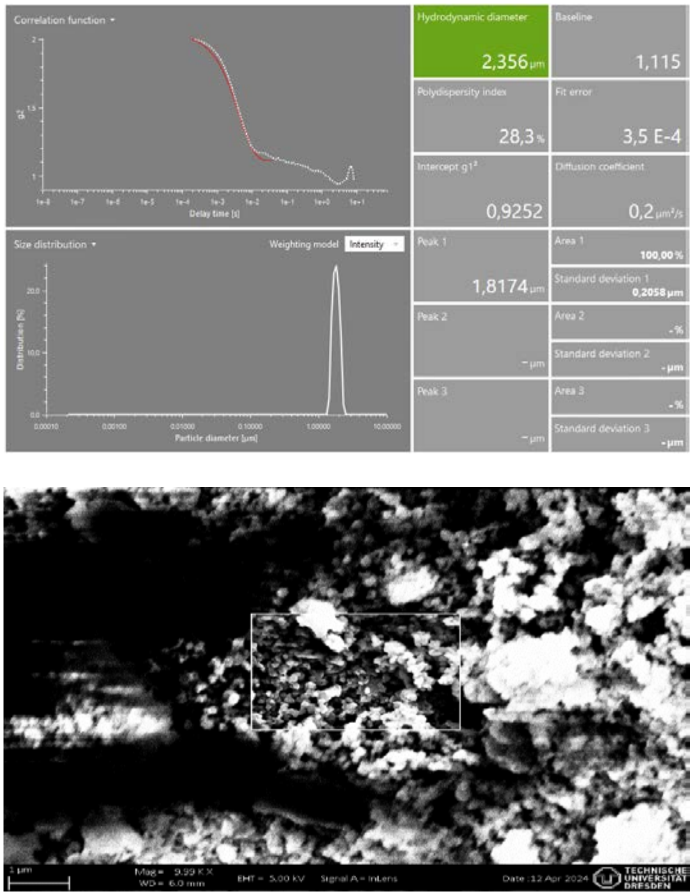

Figure SI-3: a) Hydrodynamic diameter of milled Lewatit VP OC 1600 particles analyzed via DLS. Particles were dispersed in ethanol in a polystyrene latex 2.5 mL macro PS cuvette (12.5 x12.5 x45 mm) and subjected to 60 runs per measurement. The back scatter angle was set to 175°. b) SEM-micrographs of milled Lewatit VP OC 1600 analyzed at a magnification 9.99 KX. Milling was done for 30 h at a frequency of 30 Hz on a Retsch mixer mill MM300 (Retsch, Germany).

(a) Stoeber synthesis silica

	Native	Sil-VS	Sil-VS-PEG	Sil-PEG
0 h	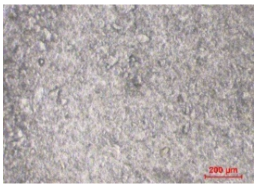	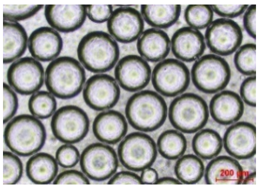	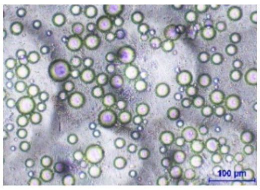	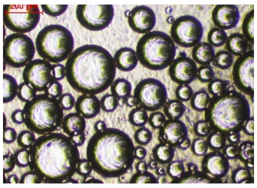
24 h	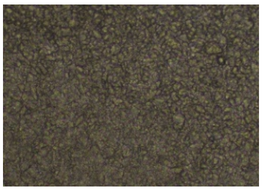	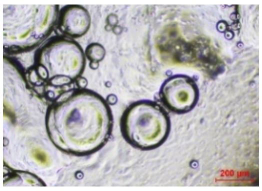	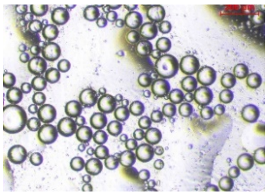	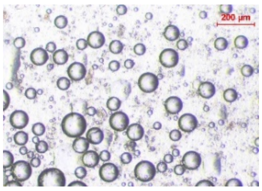
48 h			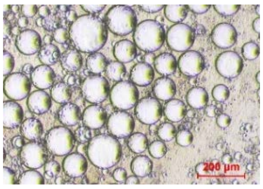	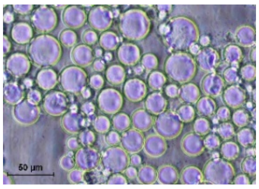

(b) Commercial silica

	Native	Sil-VS	Sil-VS-PEG	Sil-PEG
0 h	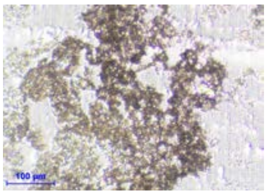	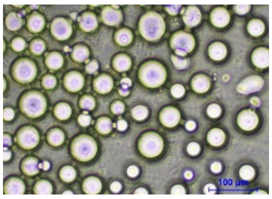	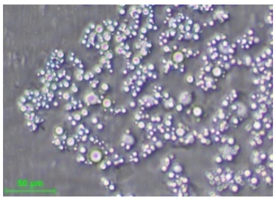	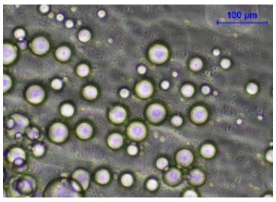
24 h	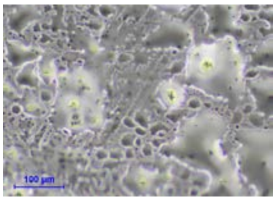	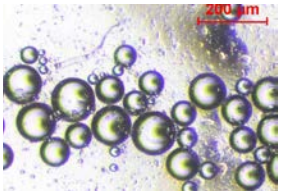	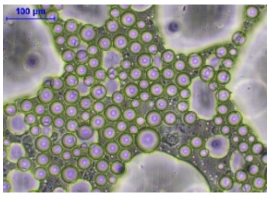	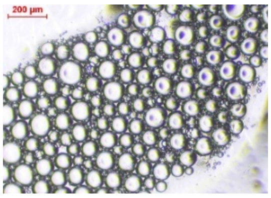
48 h		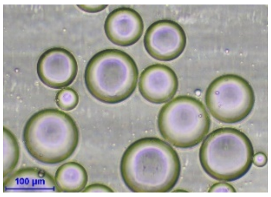	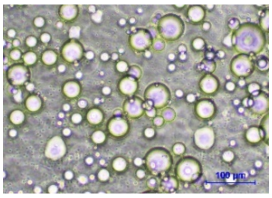	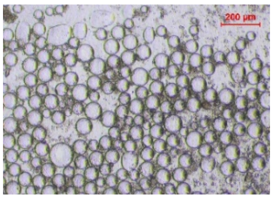

(c) Magnetic

	Native	Sil-VS	Sil-VS-PEG	Sil-PEG

0 h	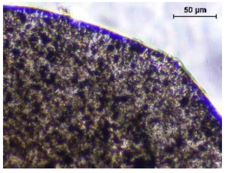	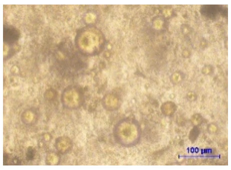	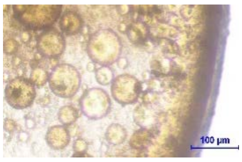	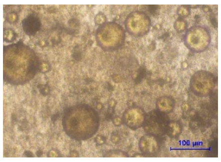
24 h		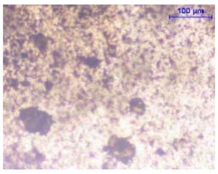	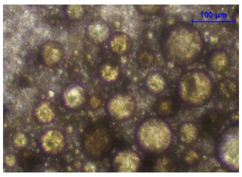	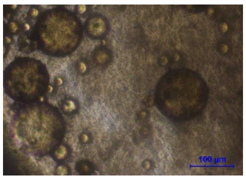
48 h			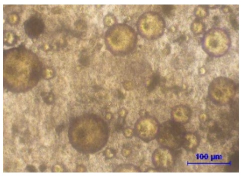	

**(d) Celite**				
	Native	Sil-VS	Sil-VS-PEG	Sil-PEG
0 h			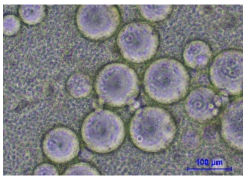	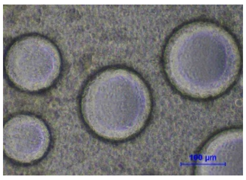
24 h			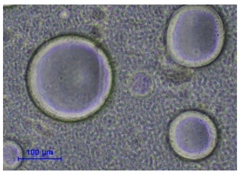	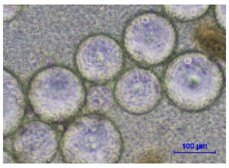

**(e) Accurel MP 1001**				
	Native	Sil-VS	Sil-VS-PEG	Sil-PEG
0 h	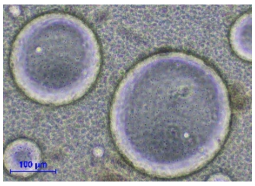	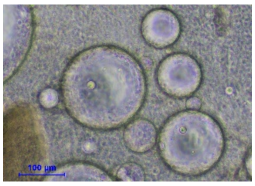	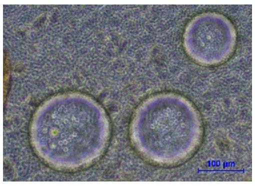	

**(f) HDK®H2000**

	Native	Sil-VS	Sil-VS-PEG	Sil-PEG
0 h	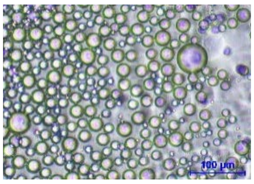	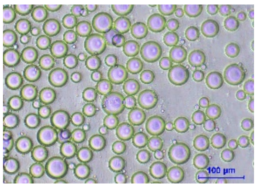	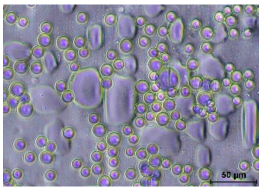	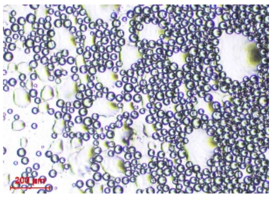
24 h	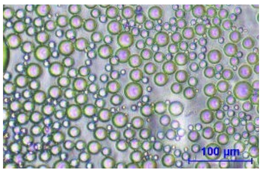	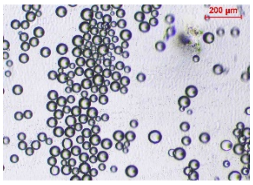	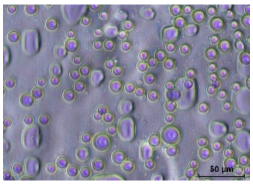	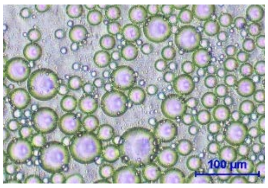
48 h	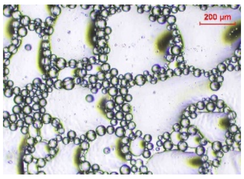	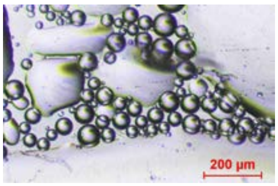	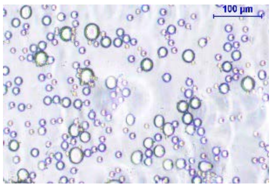	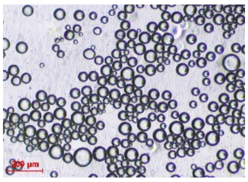

(g) Lewatit VP OC 1600

	Native	Sil-VS	Sil-VS-PEG	Sil-PEG
0 h	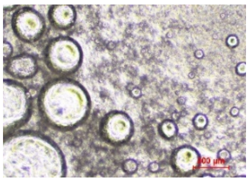	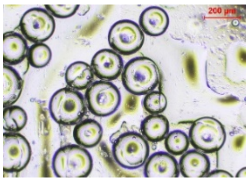	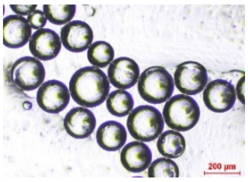	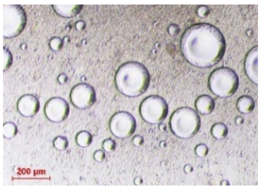
24 h	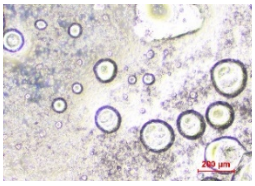	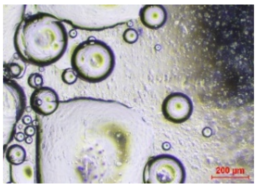	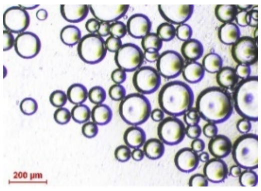	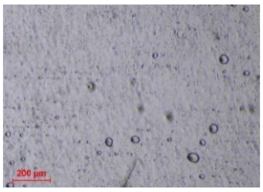
48 h	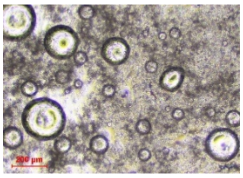	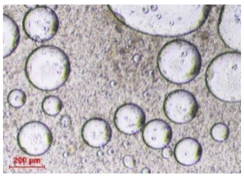	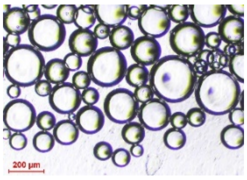	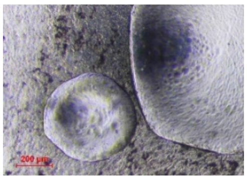

(h) Milled Lewatit VP OC 1600

	Native	Sil-VS	Sil-VS-PEG	Sil-PEG

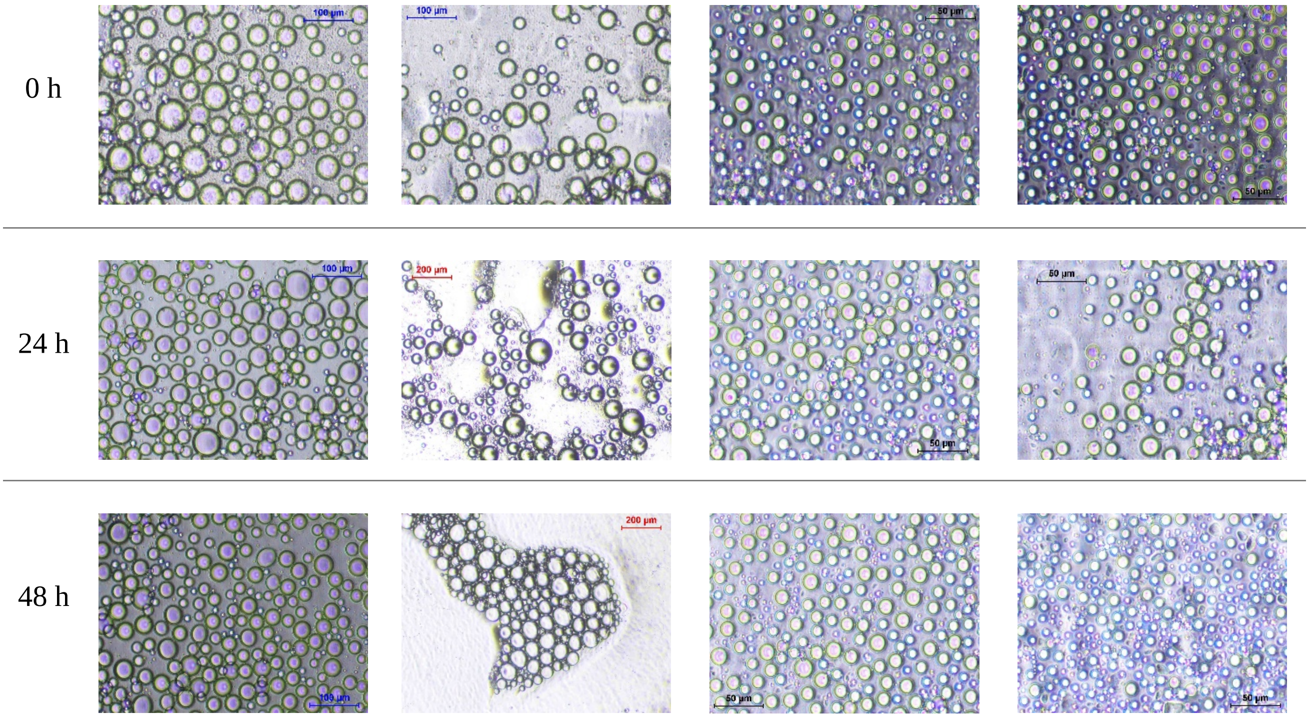

Figure SI-4: Size, morphology and distribution of droplets dispersed in w/o PE directly after formation, after 24 h and after 48 h established with native and silicone-coated material at a phase ratio of 33 % (v_{dp}/v) KPi buffer (50 mmol L^{-1}, pH 7) in CPME and a particle concentration of 30 g $L^{-1}{}_{dp.}$ (**a**) Stoeber synthesis silica, (**b**) commercial silica (Sigma, Germany), (**c**) magnetic Fe_3O_4, (**d**) Celite, (**e**) Accurel MP1001, (**f**) HDK®H2000 (**g**), Lewatit VP OC 1600, and (**h**) Lewatit VP OC 1600 after milling for 30 h at 30.1 sec^{-1} with a Retsch bead mill MM300 (3 SS and 5 SS steel beads). Betweeen measurements, emulsions were stirred at 99 rpm and images were taken using a Nikon eclipse Ts2 inverted electron microscope (Japan) at 4x, 10x and 20x magnification. The samples were diluted with ethyl acetate whenever required.

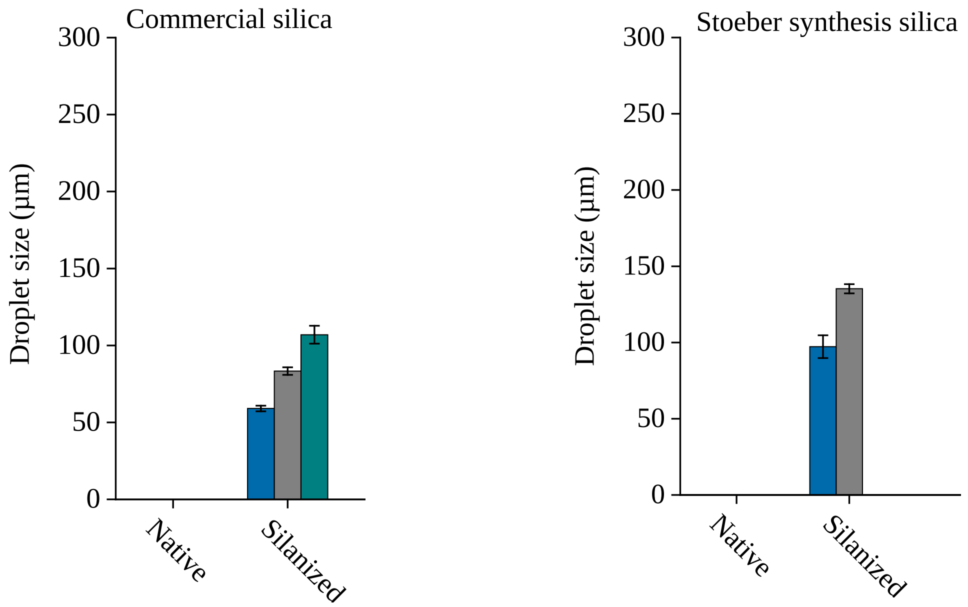

Figure SI-5: Average droplet sizes directly after formation, 24 h and 48 h of w/o PE established with (a) native and (b) silanized silica particles: Phase ratio of 33 % (v_{dp}/v) KPi buffer (50 mmol L^{-1}, pH 7) in CPME, and particle concentration of 30 g $L^{-1}{}_{dp.}$ Emulsions were stirred at 100 rpm between measurements, and error bars represent standard deviations based on three independent experiments.

Table SI-2 : Total protein loading (%) on Lewatit VP OC 1600 and Stoeber synthesis silica particles, before and after Sil-VS-PEG coating.

	**After immobilization**	**After drying**	**After Sil-VS-PEG Coating**
**Lewatit VP OC 1600**	93.25 ± 0.09	92.13 ± 0.22	86.04 ± 0.02
**Stoeber Silica**	35.065±0.02	28.35±0.03	21.98±0.02

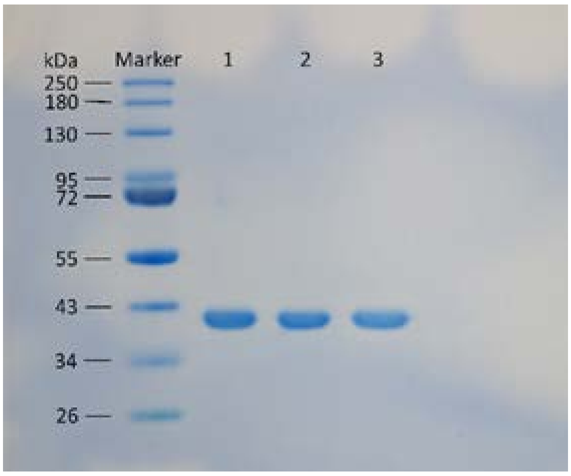

Figure SI-6: SDS-PAGE of fractions from the His-tag purification of CalB. Lane 1: Precision Plus Protein Dual Color Standard marker (Bio-Rad Laboratories GmbH, Germany); lanes 2-4: Elution fractions of purified protein. Gels were stained with Coomassie Blue G-250.

Table SI-3: Chemicals and solvents.

Name	Cas	Manufacturer
1-Phenylethanol ($C_8H_{10}O$)	98-85-1	Sigma-Aldrich (USA)
Allyl chloride (C_3H_5Cl)	107-05-1	Thermo Fisher Scientific Inc. (USA)
Ammonia 25% (NH_3)	1336-21-6	Merck KGaA. (Germany)
Ammonium hydroxide (NH_4OH)	1336-21-6	Merck KGaA. (Germany)
Cyclopentyl methyl ether (CPME) ($C_6H_{12}O$)	5614-37-9	VWR (USA)
DNAse I	9003-98	Thermo Fisher Scientific Inc. (USA)
Ethanol (C_2H_6O)	64-17-5	Carl Roth GmbH & Co. (Germany)
Hexane (C_6H_{14})	110-54-3	VWR (USA)
Imidazole	288-32-4	Carl Roth GmbH & Co. (Germany)
Iron (II) chloride tetrahydrate ($FeCl_2{\cdot}4H_2O$)	13478-10-9	Merck KGaA. (Germany)
Iron (III) chloride ($FeCl_3$)	7705-08-0	Carl Roth GmbH & Co. (Germany)
Isopropanol (C_3H_8O)	67-63-0	VWR (USA)
Methanol (CH_3OH)	67-56-1	VWR (USA)
Platinum(0)-1,3-divinyl-1,1,3,3-tetramethyldisiloxane complex solution / Karstedt catalyst ($C_8H_{18}OPtSi_2$)	68478-92-2	Thermo Fisher Scientific Inc. (USA)
Polyethylene glycol 400 (Peg400)	25322-68-3	Thermo Fisher Scientific Inc. (USA)
Sodium chloride (NaCl)	7647-14-5	Carl Roth GmbH & Co.(Germany)
Sodium hydroxide (NaOH)	1310-73-2	Carl Roth GmbH & Co. (Germany)
Tetraethoxysilane (TEOS) ($SiC_8H_{20}O_4$)	78-10-4	Sigma-Aldrich (USA)
Trimethoxy(octadecyl)silane (TMOD) ($C_{21}H_{46}O_3Si$)	3069-42-9	abcr GmbH (Germany)
Toluene ($C_6H_5CH_3$)	108-88-3	Thermo Fisher Scientific Inc. (USA)
Vinyl butyrate ($C_6H_{10}O_2$)	123-20-6	TCI GmbH (Germany)
Vinyl-terminated polydimethylsiloxane, reduced volatility (Polymer VS 50)	68083-19-2	Sigma-Aldrich (USA)
